# Structural Modifications of 3-Triazeneindoles and Their Increased Activity Against *Mycobacterium tuberculosis*

**DOI:** 10.3390/antibiotics9060356

**Published:** 2020-06-24

**Authors:** Konstantin B. Majorov, Boris V. Nikonenko, Pavel Yu. Ivanov, Lyudmila N. Telegina, Alexander S. Apt, Valeria S. Velezheva

**Affiliations:** 1 Central Research Institute for Tuberculosis, Moscow 107564, Russia; majorov@list.ru (K.B.M.); boris.nikonenko52@gmail.com (B.V.N.); 2 Institute for Element-Organic Chemistry Russian Academy of Sciences (INEOS RAS), Moscow 119334, Russia; smushk@yandex.ru (P.Y.I.); larina@ineos.ac.ru (L.N.T.); vel@ineos.ac.ru (V.S.V.)

**Keywords:** tuberculosis, drug candidates, 3-triazenoindoles

## Abstract

We synthesized 100 novel indole-based compounds with polyaza-functionalities, including 3-triazeneindoles, and tested their activity in vitro against laboratory *M. tuberculosis* H37Rv and clinical izoniazid-resistant CN-40 isolates, using gross and fine titration approaches. Here we present a few 3-triazeneindoles with the highest anti-mycobacterial activity. Introduction of short lipid tails into the 3-triazeneindole core additionally increased their activity against mycobacteria engulfed by murine macrophages. We also demonstrate that the compound TU112, one of the most active in our previous study, being not bioavailable after administration in mice *per os*, manifests prominent anti-mycobacterial activity after intravenous or aerosol delivery, as assessed by the mouse serum and lung supernatant titration assays.

## 1. Introduction

There is an urgent need for novel anti-tuberculosis (TB) drugs active against multidrug resistant (MDR) and extensively drug-resistant (XDR) *M. tuberculosis* strains [1,2]. With few exceptions, current anti-mycobacterial drugs and compounds in clinical trials are aimed at well-established critical cell syntheses: wall, essential protein, and nucleic acids (reviewed in [3]). Given their central role in general biology, there is little doubt that the majority of ongoing TB drug studies continue targeting these biochemical pathways, although the search for alternative targets is intensive (e.g., see ref. [4,5]). However, as a recent example with five newly introduced TB drugs shows, the acquisition of resistance to any compound by mycobacteria is a matter only of a relatively short time [6], so the pipeline should always be full with new candidates.

In our previous study, we showed that replacing the amino group in 3-amino-2-ethoxycarbonyl indoles with three lined-up nitrogen atoms, i.e., the triazene group, results in the creation of 2-ethoxycar bonyl-3-triazeneindoles. Among more than 200 target-synthesized indole-based compounds, seven 3-triazenoindoles displayed a good level of in vitro activity (minimal inhibitory concentration (MIC) between 0.2 and 0.5 μg/mL) against *Mycobacterium tuberculosis* strain H37Rv, isoniazid (INH)-resistant human isolate CN-40, and *Mycobacterium avium*. The compound TU112 was additionally active against an artificially created dormant *M. tuberculosis* strain. These compounds showed high selectivity indexes (SI) for *M. tuberculosis* and *M. avium* in the infected mouse macrophage model [7].

To improve the efficacy and pharmacokinetic properties of the title products, we designed and synthesized 100 novel 3-N-substituted derivatives of indole-2-carboxylic acid. Here we present a few novel indole-based compounds, with higher levels of anti-tuberculosis activity, selected from a newly synthesized panel. The activity of selected compounds against intracellular *M. tuberculosis* was further increased by substitution of the ethyl ester COOC_2_H_5_ group, at the position 2 of the indole ring, with the short-chain 1-octanol-based lipid tail COOC_8_H_17_, an octyl ester group. In addition, we addressed the problem of the low bioavailability of the previously tested compound TU112 with a high level of anti-mycobacterial activity. To this end, we tested the anti-mycobacterial activity of (i) mouse sera obtained after intravenous administration of TU112, and (ii) lung homogenate supernatants obtained after TU112 inhalation.

## 2. Results and Discussion

The anti-mycobacterial performance of newly synthetized compounds is displayed in Table 1 in numerical order. Six compounds with the highest activity (MIC = <0.021–0.4 µg/mL) are highlighted in bold. Remarkably, MICs for the INH-resistant CN-40 strain were similar. The activity of some of these compounds is higher than of those described previously [7,8], which makes these candidates attractive for further preclinical studies using a widely accepted algorithm [9].

**Figure 1:**
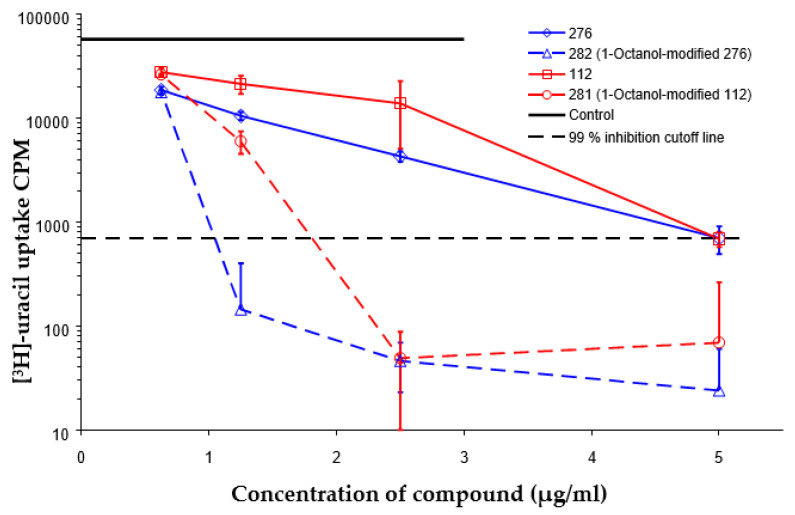
Inhibition of ^3^[H]-uracil uptake by intracellular mycobacteria. Results are expressed as medium counts per minute (CPM ± SD) for triplets. Solid black line (control): the level of [^3^H]-uracil uptake in the absence of compounds. Dashed line: the level of 99% inhibition compared to control samples.

Considering chemical modifications that might increase the performance of previously established compounds, we assumed that the introduction of a hydrophobic tail at the position 2 of the indole nucleus would increase its permeability through the macrophage cell membrane, and thus improve its bactericidal activity against intracellular mycobacteria. Figure 1 and Table 2 display the results of the testing of such modified compounds, in comparison with the parental ones. Comparison of the original “ethanol esters” TU276 and TU112 with their 1-octanol modified “octanol esters” TU282 and TU281 demonstrated that the latter were 2–4-fold more effective against macrophage-engulfed *M. tuberculosis*, despite the significant decrease of their activity against extracellular bacteria. 1-Octanol [CH_3_(CH_2_)_7_OH] itself displayed no anti-mycobacterial activity (MIC > 80 µg/mL).

These results suggest that the 1-Octanol-modified compounds behave like prodrugs, whose anti-mycobacterial activity restores and even increases inside the host cells [10].

Another problem that arose during our previous studies was the low bioavailability of the TU112 compound (the one with an appreciable in vitro anti-mycobacterial performance [7]). Assuming that its poor performance in vivo may be due to metabolic destruction after administration *per os*, we evaluated the in vitro anti-mycobacterial activity of sera and lung tissue homogenates obtained from mice that had received TU112, by, respectively, intravenous injection and inhalation. To this end, we used the inhibition titration assay, described in detail by Onajole et al. [11]. As shown in Table 3, the final dilution of mycobacteria-active sera from mice intravenously treated with TU112 was about an order of magnitude higher than that from the control mice. The final active dilution of lung homogenate supernatants from mice that received TU112 by inhalation was 8-fold higher than in controls. These results suggest that TU112 possess potential anti-mycobacterial activity in vivo.

Taken together, our results indicate that 3-triazeneindoles represent a class of compounds with an impressive activity against virulent mycobacteria, and that their chemical modification with hydrophobic tails at the indole nucleus further improves their performance against intracellular mycobacteria. Now we are starting evaluation of their activity in vivo using several mouse TB models.

## 3. Materials and Methods

### 3.1. Compounds Synthesis

A series of new 1,1-dialkyl-3-[2-(ethoxycarbonyl) indol-3-yl]triazenes, “triazene ethyl esters”, (ECIT), (TU214, 236, 250, 251, 276, 289, 300), as well as the previously described [7] compound TU112 (Table 1), incorporating N-methylpiperazine and related congeners, were synthesized as described earlier [12,13] by the N–N cross coupling reactions of appropriate 2-ethoxycarbonyl-3-diazo-3H-indoles (ECDI), at a large excess (ratio = 1:10) of appropriate piperazines or diethanolamine. A set of new 1,1-dialkyl-3-[2-(octyloxycarbonyl) indol-3-yl]triazenes (TU 281, 282, 290, 311) “triazene octyl esters” (OCIT) were synthesized analogously by the N–N cross coupling reactions of appropriate 2-octyloxycarbonyl-3-diazo-3H-indoles (OCDI) at a large excess (ratio = 1:10) of appropriate piperazines. Reactions were continued until the diazo stretch band (~2100 cm) disappeared. The yields of triazeneindoles were 30–90%. Initial 2-octyloxycarbonyl indoles required for the synthesis of OCDI were obtained by the Fisher indole synthesis followed by alkaline hydrolysis and subsequent p-toluenesulfonic acid-catalyzed esterification of appropriate indole-2-carboxylic acids with 1-octanol, as described [14]. Chemical structures of compounds were supported by elemental analyses, ¹H-NMR, ¹³C-NMR and ESI-HRMS spectral data.

All chemicals were purchased from Sigma-Aldrich and ACROS Organics at the high purity grade and used without further purification. The yields refer to purified products and were not optimized. IR spectra were run as KBr disks on an IR-Fourier-spectrometer Magna 750 IR Nicolet. ^1^H NMR (300, 400 or 600 MHz) and ^13^C NMR (75, 101 or 151 MHz) spectra were recorded using Bruker Avance^TM^ –300, Bruker Avance^TM^ –400 or Bruker Avance^TM^ –600 spectrometers. Mass spectra were recorded using Finnigan LCQ Advantage for ESI, on a Finnigan LTQ FT Ultra for HRMS (ICR) and Finnigan Polaris Q for EI. Elemental analyses were performed at the Laboratory of Microanalysis of A. N. Nesmeyanov Institute of Organoelement Compounds, Moscow. Flash Column chromatography was performed using Silica gel Merck 60 (Merck, 230 mesh).

### 3.2. Mycobacteria

*M. tuberculosis* strain H37Rv, sub-strain Pasteur, and the clinical isoniazid-resistant *M. tuberculosis* isolate CN-40 from the collection of the Central Institute for Tuberculosis (Moscow, Russia), were maintained and prepared for in vitro studies exactly as previously described [15]. Briefly, 50 µL from a thawed 10^8^ CFU/mL aliquot was added to 30 mL of Dubos broth base (BD) supplemented with 0.5% fatty acid-poor BSA (Calbiochem) and incubated for 2 weeks at 37 °C. The resulting suspension was washed two times at 3000 g, 20 min, 4 °C with Ca- and Mg-free PBS containing 0.2 mM EDTA and 0.025% Tween 80, re-suspended in PBS with 0.025% Tween 80 and filtered through a 5 μm-pore-size filter (Millipore) to remove clumps.

To estimate the CFU content in the filtrate, 20 μL from each 5-fold serial dilution was plated onto Dubos agar (BD), and the total number of micro-colonies within the spot visible on the air-dried agar was calculated under an inverted microscope (200× magnification) after being cultured for 3 days at 37 °C. The bulk of the filtered culture was stored at 4 °C, and it was found that no change in the CFU content occurred during this storage period.

### 3.3. Animals

Mice of the C57BL/6JCit (B6) inbred strain were kept under standard conditions in the Animal Facilities of the Central Institute of Tuberculosis (Cit), Moscow, in accordance with the guidelines of the Russian Ministry of Health № 755 and the NIH Office of Laboratory Animal Welfare Assurance № A5502-11. Food and water were provided *ad libitum*. All experimental procedures were approved by the CIT Bioethics Committee (IACUC). Female mice aged 2.5–3.0 months were used.

### 3.4. MIC Evaluation

In vitro MIC for each compound under study was determined using a conventional method described previously [16]. Briefly, each inoculum containing 500 H37Rv mycobacteria was cultured in the Dubos broth in a well of the round-bottom 96-well plate for 2 days at 37 °C, 5% CO_2_. Each compound from a stock solution (4 mg/mL DMSO) was serially 1.3-fold diluted in Dubos broth and added to mycobacterial cultures at gradually declining concentrations, with the final 0.1% DMSO concentration (separate experiments demonstrated no anti-mycobacterial activity of DMSO at concentrations < 2%). Plates were additionally incubated for 14 days and examined for visible mycobacterial growth on the bottom of the well. MIC was defined as the range between the lowest drug concentration completely abrogating mycobacterial growth and the highest concentration at which the growth was discerned [7,17,18]. All samples were tested twice in triplicates.

MIC for intra-macrophage mycobacteria: Peptone-elicited peritoneal macrophages from B6 mice were obtained and purified as described earlier in detail [19]. 5 × 10^4^ peritoneal macrophages were plated in wells of flat-bottom 96-well plates (Costar-Corning) in antibiotic-free RPMI-1640 medium supplemented with 10 mM HEPES buffer, 5% heat-inactivated fetal calf serum (FCS) and 2 mM L-glutamine. Cells were allowed to adhere for 2 h at 37 °C and 5% CO_2_ and thereafter infected with *M. tuberculosis* H37Rv at the MOI = 5 and incubated for 24 h. To remove extracellular mycobacteria, culture medium was aspirated, the wells rinsed once with 37 °C-heated PBS and 0.2 mL of fresh supplemented RPMI-1640 medium added. Titrated compounds were added in triplicates of each concentration for another 48 h of culturing.

Cultural supernatants were aspirated, the wells rinsed once with warm PBS, and monolayers lysed with 0.1 mL H_2_O for 10 min at room temperature. After restoring ionic balance with 0.1 mL of 2 × Dubos broth, mycobacteria released from macrophages were incubated at 37 °C, 5% CO_2_ for 90 h. 2 μCi of [^3^H]-uracil (Isotope, St. Petersburg, Russia) per well was added for the last 18 h. [^3^H]-uracil uptake by mycobacteria was measured using Wallac 1409 liquid scintillation counter (Turku, Finland). As demonstrated earlier, parallel estimations of mycobacterial CFU counts and [^3^H]-uracil uptake under identical culture conditions provided coefficients of correlation > 0.95 [19]. MIC was defined as the lowest concentration of a compound providing 99% inhibition of [^3^H]-uracil incorporation, compared to the drug-free control wells.

### 3.5. Serum and Lung Supernatant Inhibition Assay

Female B6 mice were injected intravenously with 5 mg/kg of TU112 in 0.2 mL of the water solution of alpha-tocopheryl polyethylene glycol 1000 succinate (TPGS), prepared as previously described [20,21]. At periods of 15 min, 1 h and 4 h after inoculation, blood samples were collected, and sera samples prepared and stored at −80 °C in 100 μL aliquots. Serum samples from mice that received 25 mg/kg INH and from intact mice served as controls.

To obtain lung tissue samples, 75 mg of TU112 was dissolved in 10 mL of TPGS and put into a nebulizer. Mice were put in a 4000 cm^3^ chamber, and TU112 solution was sprayed for 1 h. Mouse lungs were extracted, homogenized in 2 mL of Dubos medium and centrifuged at 2000 g. Supernatants were collected, sterilized by filtration through 2 μm filters, and stored at −80 °C in 100 μL aliquots.

To assess anti-mycobacterial activity of serum and lung tissue samples, 100 μL of *M. tuberculosis* culture in Dubos medium (10^4^ CFU/mL) was put in the wells of a 96-well plate. 100 μL of serum and supernatant samples was added to the first well, and serial 2-fold dilutions were prepared in triplicates. Plates were incubated in a CO_2_ incubator for 2 weeks, and anti-mycobacterial activity was determined as a visual lack of growth. Five female mice were used for each time point.

## Figures and Tables

**Table 1:** Anti-mycobacterial activity of new of 3-triazenoindoles (concentration range, µg/mL) *, **.

	Molecular Formula	Against H37Rv	Against CN-40
**112**	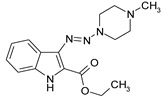	**0.19–0.25**	**0.34–0.45**
**214**	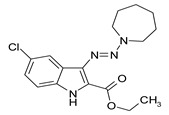	**0.095–0.13**	**0.17–0.23**
**236**	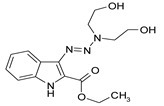	**0.021–0.028**	**0.28–0.38**
**250**	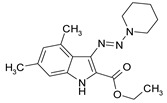	**0.071–0.095**	**0.071–0.095**
**251**	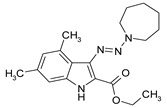	**0.071–0.095**	**0.071–0.095**
276	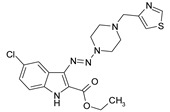	**0.19–0.25**	0.34–0.45
281	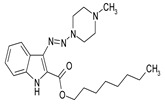	1.90–2.53	1.00–2.53
282	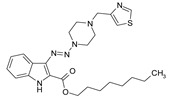	1.90–2.53	2.53–3.38
289	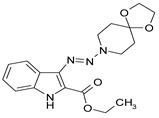	0.53–0.71	0.3–0.4
290	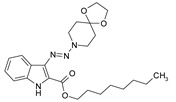	1.9–2.53	>4.5
**300**	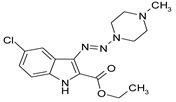	**0.3–0.4**	**0.3–0.4**
311	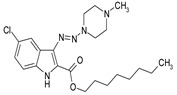	1.69–2.25	2.25–3.0

* MIC is displayed as the range between the lowest concentration for completely abrogating mycobacterial growth and the highest concentration at which the growth was discerned. ** Approved Russian patent application # 2019113030 “3-triazenoindoles active against mycobacteria”, to be issued.

**Table 2:** Introduction of octyl residues improves performance of 3-triazeneindoles against intracellular *M. tuberculosis.*

Compound	TU276	TU282 *	TU112	TU281 **
MIC for intra-macrophage mycobacteria (µg/mL)	5.0	1.25	5.0	2.5
MIC for free mycobacteria (µg/mL)	0.19–0.25	1.92–2.53	0.19–0.25	1.90–2.53

* 1-Octanol-modified TU276. ** 1-Octanol-modified TU112.

**Table 3:** Final dilutions of serum and lung homogenate samples providing inhibition of mycobacterial growth *.

Sera from Intact Mice	Sera from TU112-Treated Mice			Serum from INH-Treated Mouse	Lung Tissue from Intact Mice	Lung Tissue from TU112-Treated Mice
3.6 ± 3.0	15 min	1 h	4 h	243 ± 172	2.0 ± 1.4	17.6 ± 8.7
	64 ± 39	70 ± 35	64 ± 39			
ANOVA, *p* = 0.017					ANOVA, *p* = 0.0044	

* 5 mice per group were tested individually.
